# Diagnostic Impact of Monitoring Visual Evoked Potentials to Prevent Visual Complications During Endovascular Treatment for Intracranial Aneurysm

**DOI:** 10.3389/fneur.2022.761263

**Published:** 2022-02-23

**Authors:** Ichiro Nakagawa, HunSoo Park, Masashi Kotsugi, Shohei Yokoyama, Kouji Omoto, Kaoru Myochin, Yasuhiro Takeshima, Ryosuke Matsuda, Fumihiko Nishimura, Shuichi Yamada, Tsunenori Takatani, Hiroyuki Nakase

**Affiliations:** ^1^Departments of Neurosurgery, Nara Medical University, Kashihara, Japan; ^2^Departments of Radiology, Nara Medical University, Kashihara, Japan; ^3^Division of Central Clinical Laboratory, Nara Medical University, Kashihara, Japan

**Keywords:** visual evoked potential monitoring, coil embolization, intracranial aneurysm, visual disturbance, complications

## Abstract

**Introduction:**

The present study aimed to determine the incidence of intraprocedural visual-evoked potential (VEP) changes and to identify correlations with intraprocedural ischemic complications during endovascular treatment in patients with intracranial aneurysm related to visual function.

**Methods:**

This study analyzed data from 104 consecutive patients who underwent endovascular coil embolization to treat intracranial aneurysms related to visual function under VEP and transcranial motor evoked potential (MEP) monitoring. We analyzed associations between significant changes in MEP and VEP, defined as a >50% decrease in amplitude, and both intraprocedural complications and postoperative neurological deficits. Factors associated with postoperative neurological deficits were also assessed.

**Results:**

Treated aneurysms were predominantly located in the internal carotid artery (95%). Five (5%) were located in the posterior cerebral artery (PCA). Significant decreases in intraprocedural VEP occurred in four patients (4%), although one of those four patients did not show concomitant MEP decreases during procedures. Immediate salvage procedures avoided postoperative visual disturbances. All VEP decreases were transient and not associated with postoperative visual impairment. One of three cases who underwent intraoperative balloon occlusion test showed tolerance to balloon occlusion of the proximal PCA under VEP assessment; parent artery occlusion was performed without postoperative visual disturbance in that case.

**Conclusion:**

Although significant VEP decreases occurred 4% during neuro-endovascular aneurysm treatment related to visual function, intraprocedural VEP monitoring identifies ischemic changes associated with visual pathways and facilitates prompt initiation of salvage procedures.

## Introduction

Ischemic complications including visual disturbance are a major form of morbidity with endovascular intracranial aneurysm treatment. Stent-assisted coil embolization using neck-bridging stents and flow diverter stent placement have become standard for treating wide-necked or large aneurysms. However, stent placement is associated with increased risks of thromboembolic complications (1–3), and clinicians should pay attention to prevent ischemic complications during endovascular treatment to reduce morbidity rates. Neurophysiological monitoring with somatosensory evoked potential (SSEP) and motor evoked potential (MEP) monitoring has been applied during cerebrovascular surgery to detect functional motor and sensory disturbances (4–8). Recent studies have suggested that SSEP and MEP monitoring can reliably evaluate the ischemic status during endovascular aneurysm treatment and can improve clinical outcomes (9–12).

In contrast, preservation of visual function is also important during neurosurgical treatments closely related to the optic pathway and visual cortex. The possible utility of intraoperative visual evoked potential (VEP) monitoring has been reported for parasellar tumors and aneurysm surgery (13–16). However, no reports have described intraoperative VEP monitoring in endovascular aneurysm treatment or the efficacy of VEP assessment during the balloon occlusion test (BOT). The present study aimed to determine the incidence of intraprocedural VEP and MEP changes and whether such changes correlate with intraprocedural complications among patients after endovascular treatment of intracranial aneurysms.

## Materials and Methods

### Study Design

This retrospective observational study was based on the criteria of the STROBE (Strengthening the Reporting of Observational Studies in Epidemiology) statement. This study analyzed intraoperative VEP changes and postoperative neurological outcomes for patients who underwent endovascular coil embolization of intracranial aneurysms at a single center between 2017 and 2021. Consecutive neuro-endovascular procedures proceeded under VEP monitoring.

### Inclusion Criteria

The inclusion criteria comprised consecutive unruptured aneurysms with a diameter >5 mm, and ruptured aneurysms that were considered suitable for endovascular treatment. All patients with unruptured aneurysms were administered antiplatelet agents, aspirin (100 mg/day) and clopidogrel (75 mg/day) from 1 week before the procedure. Platelet function in all patients was analyzed using the VerifyNow Rapid Platelet Function Assay (Accumetrics, San Diego, CA, USA) 2 days before coil embolization. Patients who were resistant to clopidogrel were given adjunctive cilostazol (200 mg/day) for 2 days before the procedure. Patients with ruptured aneurysms were administered aspirin (100 mg) during the procedure. The baseline clinical characteristics collected from patient records comprised age, sex, history of risk factors, and preoperative medical conditions. Patients with diabetes mellitus were included if they had been medically managed for at least 2 months without changes in hypoglycemic treatment regimens. Chronic kidney disease (CKD) was determined as an estimated glomerular filtration rate (eGFR) <60 ml/min/1.73 m^2^. Current smokers were defined as those who had smoked at least one cigarette per day during the month before the procedure. The institutional review board at our University approved the study protocol (approval no. 2368). All patients provided written, informed consent to participate in all endovascular procedures including MEP and VEP monitoring and to allow access to their medical records for research purposes.

### Endovascular Procedures

An activated clotting time >275 s was maintained using intravenous heparin throughout coil embolization procedures under general anesthesia. To prevent mechanical vasospasm, continuous vasodilator administration was performed *via* guiding catheter. After placing a guiding catheter in the internal carotid artery (ICA) or distal vertebral artery, a microcatheter was navigated to the orifice of the aneurysm. A stent for assisted coiling was generally indicated for wide-necked aneurysms (>4 mm) or those with an unfavorable dome-to-neck ratio (<1.5), when balloon-assisted coiling failed, or when the coil protruded into the parent artery. All stents were deployed following the standard procedure, and aneurysms were sequentially coiled using detachable coils. Flow diverter stents were indicated for large aneurysms (>10 mm) at the ICA portion. Conebeam-CT images were obtained immediately after coil embolization to identify hemorrhagic changes.

### Anesthesia

Anesthesia was induced with a bolus injection of propofol (1–2 mg/kg body weight), fentanyl (2 mg/kg body weight) and vecuronium (0.1 mg/kg body weight) or rocuronium (0.5–0.6 mg/kg body weight), and maintained with 40% oxygen, propofol (2.3–3.0 g/ml by target-controlled infusion), fentanyl (total dose, 0.3–0.5 mg), and remifentanil (0.05–0.2 mg/kg/min). No muscle relaxants were used after induction of anesthesia and insertion of an endotracheal tube. The lungs were mechanically ventilated *via* the endotracheal tube to maintain the partial pressure of arterial carbon dioxide within 35–40 mmHg. Mean arterial pressure was maintained within 70–100 mmHg throughout the procedure. Core body temperature was maintained at 35.5–37.0°C (7).

### Flash Stimulus for VEP Monitoring

We used a light emitting diode (LED) light stimulator (LFS-101 II; Unique Medical, Tokyo, Japan) for VEP monitoring, inserted needle electrodes under O1, O2, and Oz (International 10–20 method) subcutaneously in the occipital region for VEP recording electrodes, inserted needle electrodes subcutaneously under the earlobe, A1, and A2 as reference electrodes, and inserted needle electrodes into the eyebrow for electroretinography (ERG). ERG and VEP potentials were recorded using an evoked potential recorder (MEB-2208; Nihon Kohden, Tokyo, Japan). Recording conditions were: light stimulus illuminance, 10,000–20,000 lx (maximum stimulus); stimulus time, 10–20 ms; stimulus frequency, 1 Hz; and number of additions, 100. ERG monitoring confirmed that flash stimuli had reached the retina, making it easy to obtain reproducible flash VEP amplitudes under general anesthesia (17).

### Transcranial Stimulation for MEP Monitoring

Transcranial electric stimulation proceeded using a Neuromaster MEE-1232 intraoperative monitoring system (Nihon Kohden). Electrical stimulation was delivered by a pair of cup electrodes (diameter, 15 mm) instead of corkscrew electrodes to avoid subcutaneous hematomas caused by dual antiplatelet therapy. Cup electrodes were attached to the scalp at the C3 and C4 positions according to the International 10–20 electroencephalography (EEG) system. Stimulation consisting of a train of five pulses was delivered with an interstimulus interval of 2 ms and a duration of 1.5–2.0 ms. Stimulus intensity was determined at the beginning of surgery and was set just supramaximal to each stimulus. An MS-120B constant current stimulator (Nihon Kohden) was initially applied up to 200 mA. When the MEP amplitude was sufficient, constant voltage stimulation was applied up to 500 V using an SEN4100 stimulator (Nihon Kohden). Compound muscle action potentials were recorded from the skin over the abductor pollicis brevis, tibialis anterior, gastrocnemius, and abductor hallucis bilaterally using disposable Vitrode V surface electrodes (Nihon Kohden). Low- and high-cut filters were set at 1–5 Hz and 2.0–3.0 kHz, respectively. Baseline MEP was recorded after the induction of anesthesia, then MEPs were evoked continuously throughout the endovascular procedure from the insertion to the removal of the femoral sheath (every 3–5 min). The amplitude of MEP was defined as the range between maximum positive and negative peaks of polyphasic waveforms. During interventions, significant changes in MEP were defined as complete disappearance of the MEP, or a >50% decrease in baseline amplitude (12).

### Balloon Occlusion Testing Under VEP Monitoring

Under general anesthesia, a SHORYU^®^ 3 mm × 5 mm balloon-microcatheter (Kaneka, Tokyo, Japan) was placed in the ipsilateral posterior cerebral artery (PCA) proximal to the aneurysm, then the balloon was inflated and complete PCA occlusion was confirmed on vertebral angiography. The BOT was performed for 15 min. During the BOT, changes in VEP, ERG, and MEP waveforms were monitored and angiographic retrograde collateral filling of the distal PCA territory was also confirmed by ipsilateral internal carotid angiography. During BOT, significant changes in VEP were defined as complete disappearance as VEP, or a >50% decrease in baseline amplitude as well.

### Postoperative Follow-Up

After the procedure, patients who did not require stent placement were maintained on a 4-week course of aspirin monotherapy (100 mg/day). If a stent was implanted, the patient was placed on a 3-month course of dual antiplatelet agents, followed by aspirin monotherapy for at least 3 months. In all patients, diffusion-weighted imaging (DWI) was performed using multisection, single-shot, spin-echo planar imaging on postoperative day 1. Ischemic lesions that arose due to procedure-related cerebral infarctions were defined as new ipsilateral hyperintense regions on DWI. Intraprocedural complications were defined as thromboembolism (including occlusion of the parent artery or its branch artery), premature aneurysm rupture, and mechanical cerebral vasospasm. Postoperative neurological deficit included new-onset visual acuity disturbance and visual field disturbance, hemiparesis or hemiplegia, sensory dysfunction, cerebellar ataxia, cranial nerve palsy, or rapid deterioration to neurological death within 24 h of the procedure. The incidence of intraprocedural VEP and MEP changes and correlations between these changes and intraprocedural complications and postoperative neurological deficits in patients undergoing endovascular intracranial aneurysm treatment were analyzed.

## Results

### Study Population

**Figure 1:**
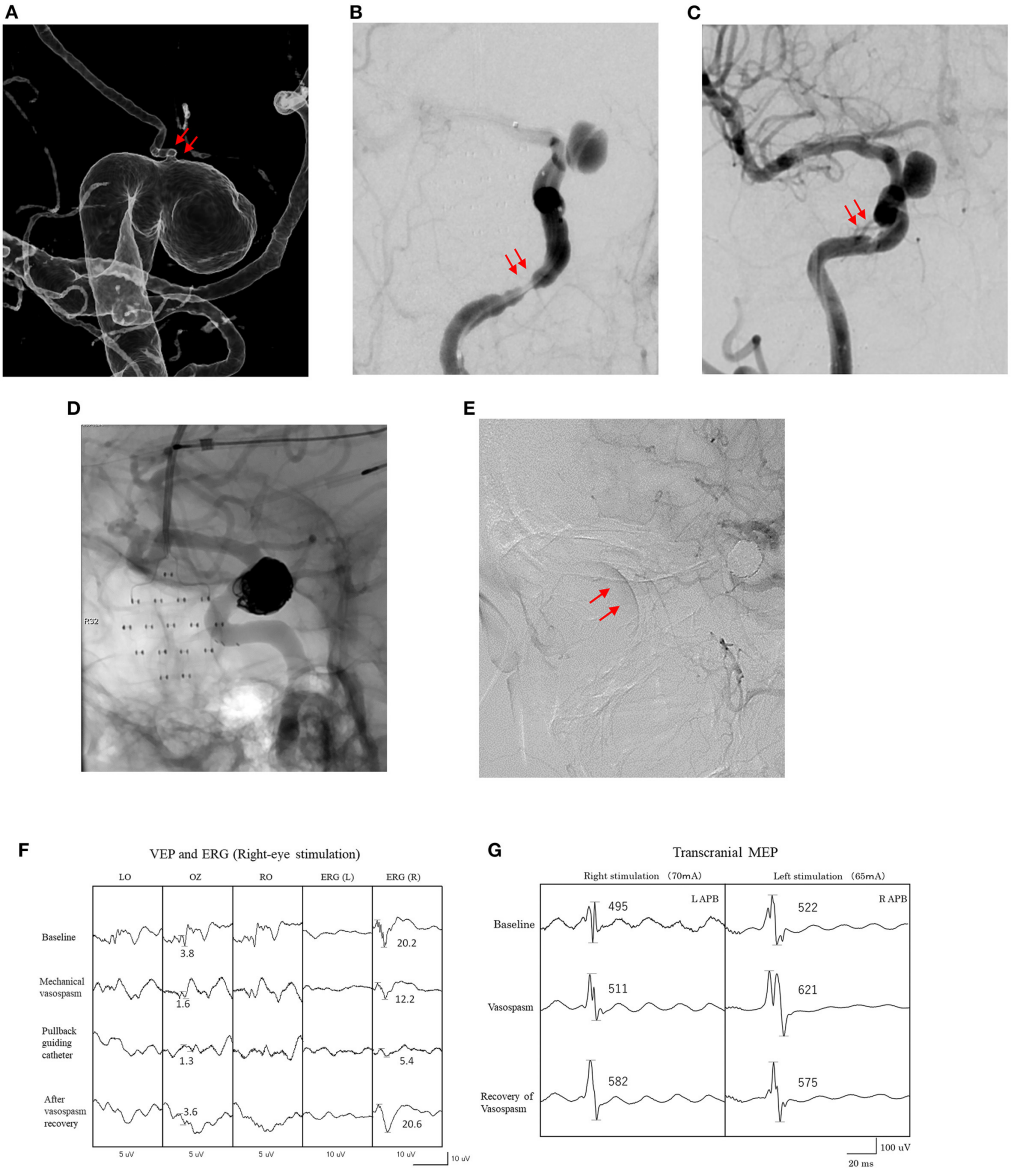
Findings for a 51-year-old woman. **(A)** Imaging shows an IC-ophthalmic aneurysm (9 mm). The ophthalmic artery originates from the dome of the aneurysm (arrows). **(B)** Mechanical cerebral vasospasm at petrous portion of the right internal carotid artery after introduction of microcatheters (arrows). **(C)** Recovery of mechanical vasospasm after pulling the microcatheters and intra-arterial administration of vasodilators (arrows). **(D)** Stent-assisted coil embolization of the aneurysm under VEP and MEP monitoring. **(E)** Retinal brush is confirmed by right internal carotid artery angiography after the procedure (arrows). **(F)** Amplitude of VEP and right ERG are decreased after onset of mechanical vasospasm. **(G)** Amplitude of transcranial MEP did not decrease during mechanical vasospasm. LAPB, left abductor pollicis brevis; RAPB, right abductor pollicis brevis.

Table 1 presents the clinical characteristics of the 104 patients (24 men, 90 women; mean age, 61 ± 14 years). Aneurysms treated under VEP monitoring were predominantly located in the ICA (95%), with the remaining located in the PCA. Stent-assisted coil embolization was applied to 77% and a flow diverter stent was applied to 16% of patients. Intraprocedural complications developed during six (6%) endovascular procedures, one of which (17%) resulted in postoperative neurological deficits. One thromboembolism due to occlusion of the ICA-anterior choroidal artery (AchA), and three extracranial ICA mechanical vasospasms arose during guiding catheter introduction. Two intraoperative ruptures included two aneurysms in the ICA-posterior communicating artery and one in the AchA. Postoperative neurological deficits developed in two patients (2%), comprising motor weakness (*n* = 1) and confusion/delirium (*n* = 1) (Table 2). No visual disturbance was encountered in this study. In one (50%) of the two patients with postoperative neurological deficits, these deficits were associated with intraprocedural complications. Postoperative DWI positivity was seen in 39% of patients and functional outcomes at discharge were good (modified Rankin Scale score 0) in 100 patients (96%) (Table 2). Figure 1 shows a representative patient.

**Table 1:** Clinical characteristics of the 104 patients.

**Characteristics**	**Value (%)**
Number of patients	104
**General characteristics**	
Age	61 ± 14
Females	90 (87%)
**Risk factor**	
Hypertension	50 (48%)
Diabetes mellitus	5 (5%)
Current smoker	12 (12%)
CKD	12 (12%)
**Medication**	
Statins	27 (26%)
ARBs	26 (25%)
PPIs	6 (6%)
DAPT	104 (100%)
**Aneurysm**	
Wide neck (>4 mm)	69 (66%)
Large size (>10 mm)	28 (27%)
ICA aneurysm	99 (95%)
IC-ophthalmic aneurysm	4 (4%)
PCA aneurysm	5 (5%)

*CKD, chronic kidney disease; ARBs, angiotensin receptor blockers; PPIs, proton pump inhibitors; DAPT, dual antiplatelet therapy; IC, internal carotid; PCA, posterior cerebral artery*.

**Table 2:** Endovascular aneurysm treatment and clinical outcomes.

**Characteristics**	**Value (%)**
Treatment	
Coil embolization	87 (84%)
Stent-assisted	80 (77%)
Flow diverter	17 (16%)
Intraprocedural complications	6 (6%)
Thromboembolism	1 (1%)
Mechanical vasospasm	3 (3%)
Minor extravasation	2 (2%)
Postprocedural DWI positivity	41 (39%)
Postprocedural neurological deficits	2 (2%)
Motor weakness	1 (1%)
Confusion/delirium	1 (1%)
Visual disturbance	0 (0%)
**Functional outcomes at discharge**	
mRS 0	100 (96%)
mRS 1	3 (3%)^*^
mRS >2	1 (1%)^**^

*mRS, modified Rankin Scale; mRS change (pre → post)*.

* *mRS 1 → 1 (n = 2), mRS 0 → 1 (n = 1)*.

** *mRS 2 → 2 (n = 1)*.

### Intraoperative VEP and MEP Changes and Postoperative Neurological Deficits

**Figure 2:**
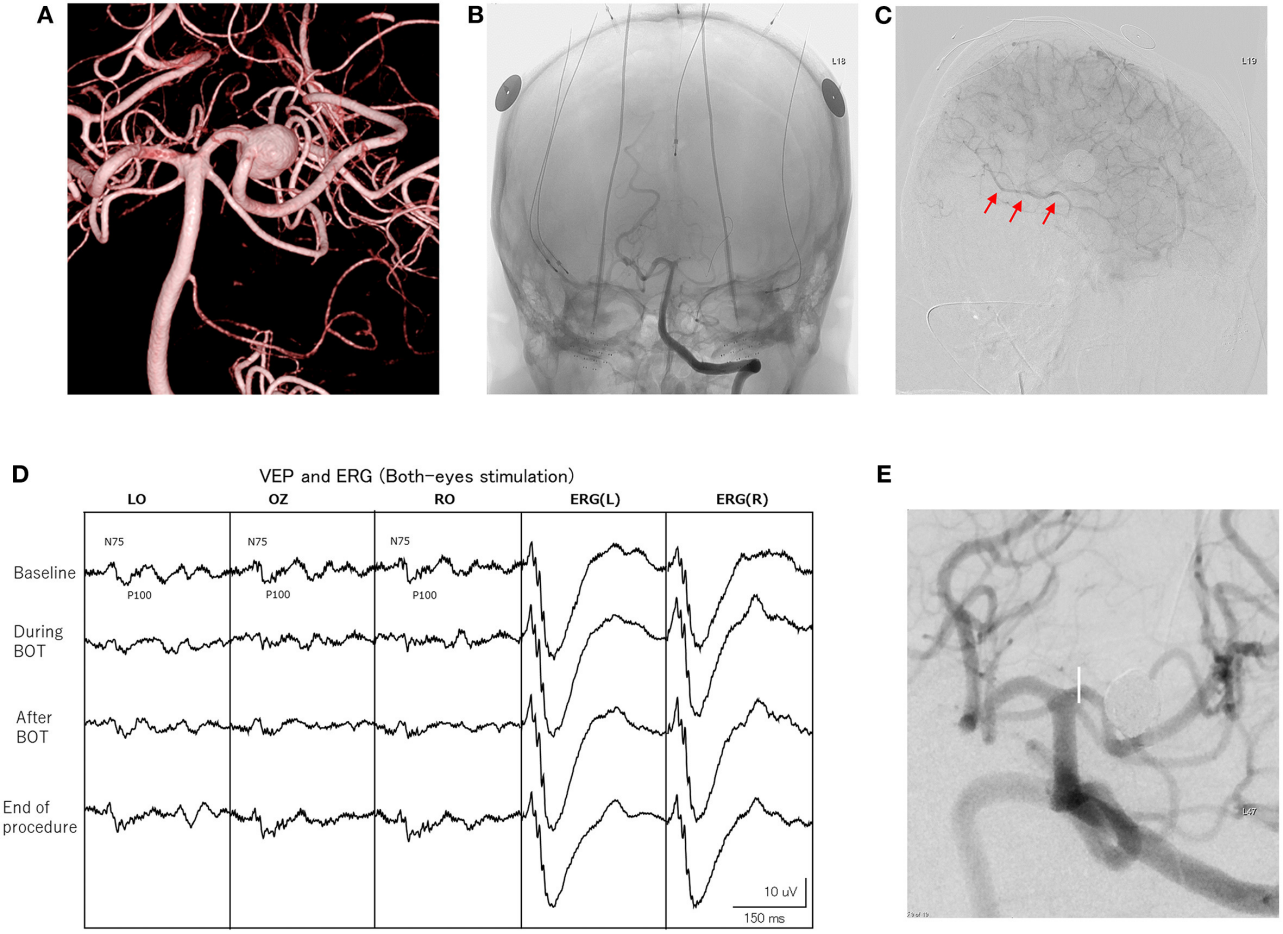
Findings for a 57-year-old woman. **(A)** Imaging shows a left large posterior cerebral artery (PCA) aneurysm (10 mm). **(B)** Left vertebral angiography depicts electrode settings for VEP, ERG and MEP monitoring. A balloon-microcatheter is positioned in the left PCA (P2A portion), then balloon occlusion test (BOT) is performed for 10 min. **(C)** During BOT, left internal carotid angiography shows retrograde collateral filling of the distal PCA territory. This is also confirmed by ipsilateral internal carotid angiography (arrows). **(D)** A significant decrease in VEP amplitude is seen during BOT. The amplitude of VEP completely recovers after the end of BOT. **(E)** Stent-assisted coil embolization of the aneurysm is performed under VEP and MEP monitoring.

Intraprocedural changes in VEP and MEP recordings were significant during five endovascular procedures (5%) (Table 3). One patient showed decreases in all three amplitudes caused by mechanical vasospasm. One patient showed only a decrease in VEP amplitude caused by mechanical vasospasm. Two transient MEP changes were caused by two balloon-inflation procedures for intraprocedural aneurysm rupture. One case with permanent MEP change involved the one AchA aneurysm. All five patients who presented with decreases in VEP, EGR, or MEP amplitude underwent immediate salvage procedures, including balloon deflation, guiding catheter repositioning, intraarterial antithrombotic drug injection and coil retrieval. The amplitudes of these values in four patients with transient changes recovered to baseline within 10 min of starting salvage procedures, and none of these four patients developed postoperative neurological deficits. In contrast, the one patient with permanent MEP changes developed postoperative neurological deficits with modified Rankin Scale score at discharge ≥1 despite salvage procedures (Table 3). False-negative and false-positive VEP or MEP changes were not encountered during endovascular procedures. Intraoperative BOTs under VEP and MEP monitoring were performed for PCA aneurysms, and two patients showed transient decreases in VEP amplitude without ERG decreases during BOT. One patient did not show decreased VEP amplitude with good retrograde collateral flow on angiography during BOT and parent artery occlusion was performed without postoperative ischemic findings (Table 4). Figure 2 shows a representative patient who underwent intraoperative BOT.

**Table 3:** Summary of patients with intraprocedural VEP and MEP changes and postprocedural neurological deficits.

**Case**	**Age**	**Gender**	**Location**	**Adjunctive**	**VEP decrease**	**ERG decrease**	**MEP decrease**	**Post-deficits**	**Causative events**	**Post-MRI findings**	**mRS at discharge**
1	70	F	Rt. ICA C2	SAT	>50% (trans.)	>50% (trans.)	>50% (trans.)	None	Mechanical vasospasm	None	0
2	51	F	Rt. IC-OphA	SAT	>50% (trans.)	None	None	None	Mechanical vasospasm	None	0
3	47	F	Rt. IC-PcomA	SAT	None	None	>50% (trans.)	None	Intraoperative rupture	Single spot in deep white mater	0
4	45	F	Rt. IC-AchA	SAT	None	None	>50% (trans.)	None	Intraoperative rupture	None	0
5	68	M	Rt. IC-AchA	SAT	None	None	>50% (permanent)	Motor weakness	Embolism	Small embolism of internal capsule	1

*OphA, ophthalmic artery; PcomA, posterior communicating artery; AchA, anterior choroidal artery; SAT, stent-assisted embolization; mRS, modified Rankin Scale; trans., transient*.

**Table 4:** Intraoperative VEP changes under balloon occlusion test for PCA aneurysms.

**Case**	**Age**	**Gender**	**Location**	**Aneurysm size**	**Anesthesia**	**Balloon size**	**BOT location**	**VEP decrease**	**ERG decrease**	**Collateral flow**	**Treatment**	**Post-deficits**
1	64	F	Rt. PCA	5.0 mm	General	3 mm × 5mm	P2A	>50% (transient)	None	poor	SAT	None
2	17	F	Lt. PCA	33 mm	General	3 mm × 5 mm	P2A	None	None	well	PAO	None
3	57	F	Lt. PCA	9.8 mm	General	3 mm × 5 mm	P2A	>50% (transient)	None	poor	SAT	None

*VEP, visual evoked potential; BOT, balloon occlusion test; PCA, posterior cerebral artery; SAT, stent-assisted embolization; PAO, parent artery occlusion*.

## Discussion

Thromboembolism is the major adverse event associated with endovascular intracranial aneurysm treatment (1, 18). Some acute or delayed ischemic complications have been reported because of the coverage of side branches by neck-bridging or flow diverter stents, especially involving the ophthalmic artery and AchA (19, 20), and visual impairments are often observed after such endovascular procedures (21, 22). Neurophysiological monitoring of SSEP and MEP in neuro-endovascular treatment has proven that SSEP and MEP monitoring during endovascular aneurysm treatment can reliably assess pyramidal tract function and improve clinical outcomes (12, 18, 23). The possible efficacy of intraoperative VEP monitoring has been reported for parasellar, temporal, parietal, and occipital brain tumors, allowing at minimum the detection of new quadrantanopia (13, 24, 25). However, few clinical studies have been published on the use of intraoperative VEP monitoring to avoid visual impairments in neuro-endovascular aneurysm treatment. This study of 104 consecutive patients with intracranial ICA and PCA aneurysms treated by stent-assisted coil embolization or flow diverter placement under VEP and MEP monitoring found intraprocedural endovascular complications in six patients (6%), with five of the six procedures (83%) avoiding postoperative neurological deficits thanks to immediate salvage procedures. All four ICA-ophthalmic artery aneurysms were treated endovascularly under VEP and MEP monitoring in this study and showed no postoperative neurological deficits.

Mechanical vasospasm can be induced by distally introducing the microcatheters for adjunctive coil embolization and the flow restriction causes thromboembolism and/or hemodynamic insufficiency, resulting in ischemic injury (26). Distally introduced, wedged catheters caused VEP/MEP waves to disappear in two patients in this study (Table 3). Interestingly, one of those two patients who showed intraprocedural mechanical vasospasm also displayed concomitant decreases in VEP and MEP amplitudes during mechanical vasospasm, whereas the other showed only a decrease in VEP amplitude (Figure 1). These differences in the response of VEP and MEP to mechanical vasospasm may be caused by differences in the development of collateral blood flow *via* anterior and posterior communicating arteries and suggested that MEP alone is not always sufficient to detect visual impairment due to hemodynamic impairment caused by mechanical vasospasm. Indeed, digital subtraction angiography (DSA) is the gold standard for detecting intraprocedural complications, since changes in the vascular anatomy can be immediately visualized during endovascular treatment. However, microembolisms in the central retinal artery in the absence of ophthalmic artery occlusion cannot be detected on DSA (21). VEP monitoring can evaluate physiological visual function independently of vascular anatomical abnormalities, providing an important method for monitoring neuro-endovascular aneurysm treatment. Salvage procedures, including balloon deflation, guiding or repositioning of microcatheters, intraarterial antithrombotic or vasodilator drug injection, and coil or stent retrieval, should be started as soon as possible to prevent permanent visual impairment.

In contrast, PCA aneurysms are relatively rare and direct surgical treatments including neck clipping and trapping with cerebral revascularization are often complicated and associated with high morbidity rates (27, 28). Endovascular treatment with stent-assisted coil embolization and flow diverter placement with preservation of the parent artery has been selected as a first choice, but these technique seem to encounter difficulty in achieving cure, particularly in cases of giant, fusiform, or partially thrombosed PCA aneurysm (29, 30). Endovascular parent artery occlusion (PAO) represents an alternative treatment strategy for such complicated aneurysms, but preoperative evaluation of ischemic tolerance is required and BOT is usually performed. Various modalities including observation of the neurological symptoms, identification of cerebral blood flow changes on imaging modalities such as CT angiography, single photon emission computed tomography or positron emission tomography, and the presence or absence of abnormal EEG have been used for evaluating the BOT (31–33). However, reliable assessment of ischemic tolerance has not been standardized (29, 34). Recently, reproducible and stable VEP monitoring has become available using a photostimulator with a high-brightness LED and using ERG monitoring under total intravenous anesthesia with propofol, which shows little suppressive effect on VEP (17, 35). In this study, stable VEP waveforms were recorded during not only BOT, but throughout the endovascular aneurysm treatment procedures, including PAO, and preservation of postoperative visual function was achieved (Table 4). Furthermore, VEP monitoring can be performed in the endovascular suite even under general anesthesia, and can evaluate waveform changes in real time.

Some limitations must be pointed out in this study. First, our study was limited by the retrospective design and limited number of cases. Second, the onset of quadrantanopia or preoperative visual impairment may not be detected by changes in VEP waveforms (24). Third, the appearance of false-positive results associated with the depth of anesthesia and displacement of the light stimulation pad, and the setting of the optimal alarm point have not been resolved (15, 17). However, VEP monitoring may offer an alternative for assessing ischemic tolerance due to its simplicity and non-invasiveness. Further clinical studies are necessary to validate the effectiveness and safety of VEP monitoring during neuro-endovascular procedures.

## Conclusions

Although significant VEP decreases occurred 4% during neuro-endovascular aneurysm treatment related to visual function, intraprocedural VEP monitoring reliably identifies ischemic changes associated with visual pathways and can facilitate prompt initiation of salvage procedures.

## Data Availability Statement

The raw data supporting the conclusions of this article will be made available by the authors, without undue reservation.

## Ethics Statement

The institutional review board at Nara Medical University approved the study protocol (approval no. 2368). The patients/participants provided their written informed consent to participate in this study.

## Author Contributions

IN, MK, and HP: conception and design or analysis and interpretation of data, or both. IN, HP, MK, SYo, KO, KM, YT, RM, FN, SYa, TT, and HN: drafting of the manuscript or revising it critically for important intellectual content. IN and HN: final approval of the manuscript submitted. All authors contributed to the article and approved the submitted version.

## Conflict of Interest

The authors declare that the research was conducted in the absence of any commercial or financial relationships that could be construed as a potential conflict of interest.

## Publisher's Note

All claims expressed in this article are solely those of the authors and do not necessarily represent those of their affiliated organizations, or those of the publisher, the editors and the reviewers. Any product that may be evaluated in this article, or claim that may be made by its manufacturer, is not guaranteed or endorsed by the publisher.
